# Domain Heterogeneity in Radiofrequency Therapies for Pain Relief: A Computational Study with Coupled Models †

**DOI:** 10.3390/bioengineering7020035

**Published:** 2020-04-07

**Authors:** Sundeep Singh, Roderick Melnik

**Affiliations:** 1 MS2Discovery Interdisciplinary Research Institute, Wilfrid Laurier University, 75 University Avenue West, Waterloo, ON N2L 3C5, Canada; rmelnik@wlu.ca; 2 BCAM—Basque Center for Applied Mathematics, Alameda de Mazarredo 14, E-48009 Bilbao, Spain

**Keywords:** radiofrequency therapies, pain relief, bioheat transfer, coupled thermo-electric analysis, multiscale models for biological tissues, feedback control systems, AI and machine learning algorithms, finite element method, coupled mathematical models, clinical applications of computational modeling

## Abstract

The objective of the current research work is to study the differences between the predicted ablation volume in homogeneous and heterogeneous models of typical radiofrequency (RF) procedures for pain relief. A three-dimensional computational domain comprising of the realistic anatomy of the target tissue was considered in the present study. A comparative analysis was conducted for three different scenarios: (a) a completely homogeneous domain comprising of only muscle tissue, (b) a heterogeneous domain comprising of nerve and muscle tissues, and (c) a heterogeneous domain comprising of bone, nerve and muscle tissues. Finite-element-based simulations were performed to compute the temperature and electrical field distribution during conventional RF procedures for treating pain, and exemplified here for the continuous case. The predicted results reveal that the consideration of heterogeneity within the computational domain results in distorted electric field distribution and leads to a significant reduction in the attained ablation volume during the continuous RF application for pain relief. The findings of this study could provide first-hand quantitative information to clinical practitioners about the impact of such heterogeneities on the efficacy of RF procedures, thereby assisting them in developing standardized optimal protocols for different cases of interest.

## 1. Introduction

Globally, pain management is an enormous challenge that places significant physical, social and economic burdens on society. In accordance with the International Association for the Study of Pain (IASP), pain is defined as “an unpleasant sensory and emotional experience associated with actual or potential tissue damage, or described in terms of such damage.” Furthermore, pain is always a highly subjective and integrative experience that is associated with biological, psychological, and social factors. This complex definition of pain continues to evolve with advancements in medical science and technology [1]. As per the current definition of pain, a plethora of pain and pain states exist, such as nociceptive pain, neuropathic pain, acute pain, chronic pain, etc. Developing an effective treatment option for tackling acute and chronic pain is the main focus of pain management therapists, owing to the significant effects they have on the quality of life of patients, including disability, mood swings, anxiety, and overuse of medicine, to name a few [2]. Notably, acute pain lasts for less than three days and tends to diminish afterward with the passage of time as healing occurs, whereas chronic pain is the extension of acute pain that can go well beyond the expected healing duration post-injury or surgery and typically lasts for more than three months, and in some cases may last indefinitely [1,2]. A multitude of drug-based and non-drug based options exists for the management of pain, which often utilizes multimodal and multidisciplinary approaches, viz., pharmaceutical, physical therapy, rehabilitation and surgery [1,2,3,4].

Chronic pain is one of the major public health problems affecting billions of people all around the world. In Canada, for example, chronic pain imposes a significant burden on healthcare resources, accounting for approximately $7.2 billion annually [5,6]. There has been widespread reliance on the usage of opioids as a pain medication for mitigating chronic pain, which can do more harm than good [7]. Several inexpensive alternative treatment options have also been explored in clinical practices for mitigating chronic pain and minimizing the usage of opioids. Among the available treatment modalities for chronic pain relief, minimally invasive radiofrequency ablation offers several advantages, such as it is precise, reproducible, cheap and highly effective [8,9]. The application of radiofrequency (RF) has been growing rapidly and increasing in popularity for treating different types of pain, such as in the management of low back pain, knee pain, hip pain, migraine, etc. [10,11,12,13,14,15,16,17]. Generally, the power delivery during such pain management procedures is done using either a continuous or a pulsed mode [14]. In the conventional continuous power delivery mode, RF currents applied between the electrode (accurately placed on the target nerve) and the dispersive ground electrode (placed on the patient’s skin) results in temperatures above 50 °C. The high temperature obtained during the continuous RF procedure results in coagulative necrosis of the neural tissue that further leads to protein denaturation and axon destruction, which ultimately stops the transmission of nociceptive signals from the periphery, thereby mitigating pain. In contrast to the continuous RF mode, which relies on the high temperature to cause neural ablation, the pulsed delivery mode utilizes the application of short pulses of RF current to the neural tissue from the RF generator that is followed by silent phases to allow time for heat dissemination [14,18,19]. The electric field generated due to these applied pulses disrupts the functioning of neuronal membrane, along with genetic changes that affect cytokine release [8]. Thus, the mechanism of action of both these modes is quite different. Importantly, the pulsed mode is a theoretically nonablative procedure, since the maximum temperature during such procedures is usually not allowed to exceed 42 °C, thus making it less destructive in comparison to the continuous RF. Although the exact explanation of the complete mechanisms of action involved during the pulsed RF procedure for treating chronic pain remains elusive, extensive research is being undertaken to quantify its associated effects [13,20]. Despite the increasing use of various radiofrequency therapies in clinical practices for treating pain, controversy still exists over their efficacy and treatment outcomes [6]. 

Computational modeling and simulations are vital tools for providing a quick, convenient and inexpensive evaluation of the treatment outcomes of such therapies, including the thermal ablative procedures. Computational modeling has been used in the past at different stages of thermal ablative procedures, which include the design and development of new protocols, as well as optimization and improvement of existing protocols of clinical systems [21,22,23,24,25,26]. Computational models also serve as a means of understanding the interaction between various physical phenomena and the effects of various intrinsic and extrinsic factors on the treatment outcomes of RF-based clinical techniques. The application of computational tools is widespread among different thermal ablative modalities, including those for treating tumors, but very few computational studies have been reported in the literature for treating pain [27,28,29]. Moreover, most of the computational studies reported to date have been conducted considering homogeneous tissues. The latter assumption means that although the ultimate goal of such works was to attain the ablation of the target nerve, in reality, its incorporation into the computational domain has been routinely neglected. Although the previously reported computational studies on the RF application for chronic pain management have addressed several important concerns and advanced this field of research by providing a quantitative prediction of the electrical field, the evaluation of the thermal field and ablation volume without accounting for heterogeneity within the computational domain, typically results in severe inaccuracies being introduced into the associated models.

Thus, the present study aims at quantifying the effects of heterogeneity in the computational domain of interest on the characteristics obtained during radiofrequency therapies for treating pain, exemplifying our main results for the ablation volume characteristics in the continuous RF procedure. The present paper has been originally initiated from a presentation of its part at the International Conference on Bioinformatics and Neurosciences (ICoBN 2019) held in Vancouver, Canada, August 26–28, 2019 [30]. As part of our comprehensive analysis that develops and extends the idea of [30], three different computational domains were considered: (a) a completely homogeneous domain comprised of muscle tissue alone, (b) a heterogeneous domain comprising of nerve embedded within the muscle tissue, and (c) a heterogeneous domain comprising of target bone, nerve and muscle tissues. Furthermore, a comparative study of continuous RF was conducted with and without utilizing a temperature-controller. This is an automated control loop proportional-integral-derivative (PID) controller within the computational domain that continuously modulates the applied voltage to keep the maximum temperature below the predefined value to avoid the occurrence of charring at the electrode tip. 

## 2. Materials and Methods 

This section provides the details of the computational domain, governing equations for the coupled thermo-electric model of the RF procedure, numerical setup and modeling details, along with the main material properties and thermo-electric characteristics considered in the present study. The details of the fidelity and integrity of the developed computational model are also provided in this section by comparing the predicted treatment outcomes from the present model and previously reported studies available in the literature.

### 2.1. Computational Domain

**Figure 1:**
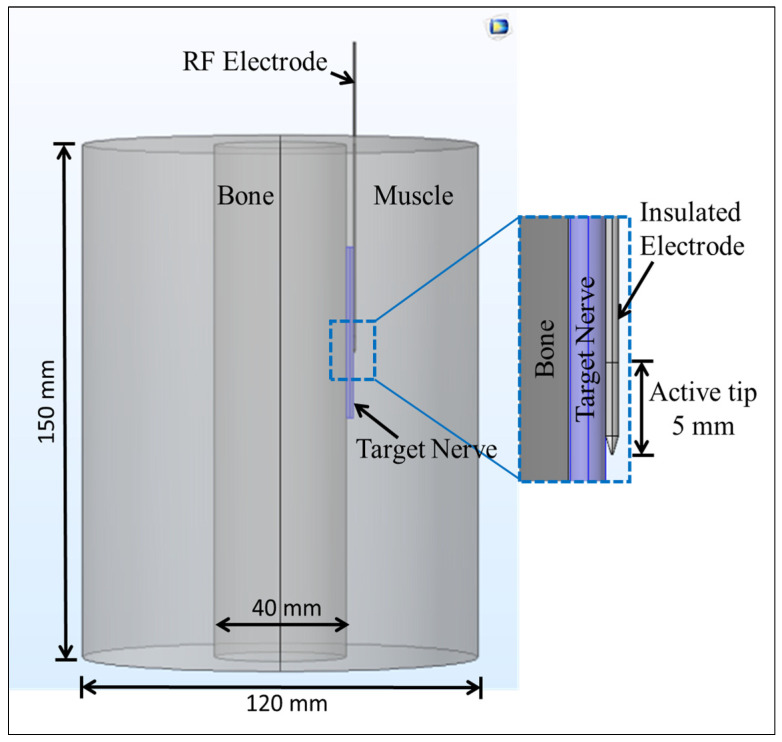
Schematic of three-dimensional computational domain comprising of nerve, bone and muscle tissues with an inserted monopolar radiofrequency (RF) electrode.

A schematic of a three-dimensional heterogeneous computational domain comprising of muscle, bone and nerve tissue [31], with an embedded 22-gauge monopolar RF electrode with a 5 mm active tip length [27] is shown in Figure 1. As mentioned earlier, the effect of heterogeneity on the efficacy of continuous RF procedure was quantified considering three different cases for the computational domain: (a) a complete homogeneous computational domain comprising of only muscle tissue, (b) a computational domain comprising of a 4 mm cylindrical nerve embedded within the muscle tissue, and (c) a completely heterogeneous computational domain comprising of bone, nerve and muscle tissues. Further possible advancements of the proposed model for case (c) are discussed later in the paper. The thermo-electric and biophysical properties considered in the present study are summarized in Table 1 [27,29,31,32]. In the present analysis, the thermo-electric characteristics of the bone presented in Table 1 correspond to the cortical bone (see Section 4.2 for additional details pertinent to this and other tissues considered here).

### 2.2. Governing Equations for Coupled Thermo-Electric Model

The RF procedure can be modeled as a coupled electro-thermal problem whereby electromagnetic energy is used to heat the biological tissue. In the lower frequency range of 500 kHz, as is generally used during RF procedures, a simplified version of Maxwell’s equations (known as the quasi-static approximation) can be used for computing the electric field distribution within the biological tissue without compromising accuracy. It is given by:

(1) ∇·(σ(T)∇V)=0

where *σ* is the temperature-dependent electrical conductivity (S/m), which has been modeled as a linear (+ 2% per °C) function of temperature in the present study [33,34,35], and *V* is the electric potential (V) that is related to the electric field “**E**” (V/m) by the standard potential field approximation given by:

(2) E=−∇V

Further, the current density “**J**” (A/m^2^) can be derived from the electrical conductivity and field as follows:

(3) J=σ(T)E

The volumetric heat generated, *Q_p_* (W/m^3^), within the biological tissue by electromagnetic field during the RF procedure is given by:

(4) $$Q_{p}=J\cdot E$$

The Fourier-conduction-based Pennes bioheat transfer equation was used for computing the temperature distribution within the biological tissue during the continuous RF procedure for pain relief. It is given by:

(5) $$\rho c\frac{\partial T}{\partial t}=\nabla \cdot \left(k\nabla T\right)-\rho_{b}\;c_{b}\omega_{b}\left(T-T_{b}\right)+Q_{m}+Q_{p}$$

where *ρ* is the density (kg/m^3^), *c* is the specific heat (J/(kg·K)), *k* is the thermal conductivity (W/(m·K)), *ρ_b_* is the density of blood, *c_b_* is the specific heat capacity of the blood, *ω_b_* is the blood perfusion rate (1/s), *T_b_* is the blood temperature (37 °C), and *T* is the unknown temperature within the biological tissue to be computed from Equation (5). The term $\rho_{b}\;c_{b}\omega_{b}\left(T-T_{b}\right)$ accounts for the heat sink effect caused by small capillary vasculature, *Q_p_* is the volumetric heat source (W/m^3^) quantified using Equation (4), *Q_m_* is the metabolic heat generation (W/m^3^), which was neglected in the present study due to its insignificant contribution as compared to *Q_p_* [27], and *t* (s) is the duration of the continuous RF procedure.

Further, in the present computational study, the blood perfusion rate was modeled utilizing a temperature-dependent piecewise model. Accordingly, a constant predefined value of blood perfusion rate prevails below the tissue temperature of 50 °C and ceases beyond that owing to the collapse of microvasculature [31], and is given by:

(6) $$\omega_{b}(T)=\left\{\begin{array}{l} \omega_{b,0}\;\;\;\;\;\;\mathrm{for}\;T50{}^{\circ}C\\ 0\;\;\;\;\;\;\;\;\;\mathrm{for}\;T\geq 50{}^{\circ}C \end{array}\right\}$$

where *ω*_b,0_ is the constant blood perfusion rate of the tissue domain given in Table 1 and *T*, as before, is the unknown temperature computed from Equation (5).

The ablation volume ($\dot{V}$) was computed using the isotherm of 50 °C (i.e., the volume of tissue having a temperature ≥ 50 °C post-RF procedure) [31] within the computational domain, and it is given by:

(7) $$\dot{V}={∭}_{\Omega }dV\;({\mathrm{mm}}^{3})\;\;\;\;\;\;(\mathrm{where}\;\;\Omega \geq 50{}^{\circ}C)$$

The continuous RF procedure was modeled by applying a constant voltage (*V*) of 15 V with a reference impedance (*Z*_ref_) of around 50 ohms, resulting in the constant power (P = *V*^2^/*Z*_ref_) of 4.5 W [36]. Further, the temperature-controlled RF procedure was performed utilizing a closed-loop feedback PID controller that varies the applied input voltage to keep the target temperature below the pre-set temperature of 85 °C, and is given by:

(8) $$V(t)=K_{p}e(t)+K_{i}\int_{0}^{t}e(\tau )d\tau +K_{d}\frac{d}{dt}e(t)$$

where *V*(t) is the applied voltage (V) during the RF procedure for mitigating pain, *e* is the error, *t* is the treatment time (s) and *K*_p_ (= 0.02), *K*_i_ (= 0.01) and *K*_d_ (= 0.001) are the proportional, integral and derivative gains, respectively [33,37,38].

### 2.3. Numerical Setup and Modeling Details

The coupled thermo-electric models of continuous RF application for treating pain were solved by utilizing the finite element method (FEM), and implemented in the COMSOL Multiphysics 5.2 software [39] using an adaptive time-stepping scheme. The continuous RF procedures were performed by applying a constant voltage of 15 V at the active tip of the RF electrode. Further, the dispersive (ground) electrode was modeled by applying 0 V on the outer boundaries of the computational domain. The initial voltage and temperature prior to the application of the RF procedure within the entire computational domain were considered to be 0 V and 37 °C, respectively, and the thermal and electrical continuity boundary conditions were imposed at each interface. Additionally, the computational model for temperature-controlled RF was developed utilizing an automatic PID controller [33,34,35] to limit the maximum temperature to 85 °C and its treatment outcomes were compared to the model with a constant voltage source of 15 V. In its essence, we are dealing here with a feedback control system, and we used this controller to improve the system by combining its closed-loop control with the open-loop feedback control. This compensates for the difference (error) between the set-point and the system response to the feedback control. As a result, the system can, in principle, utilize artificial intelligence (AI) and machine learning algorithms [40,41,42,43,44]. In the present computational study, the treatment time of the continuous RF procedure was set to 60 s.

**Figure 2:**
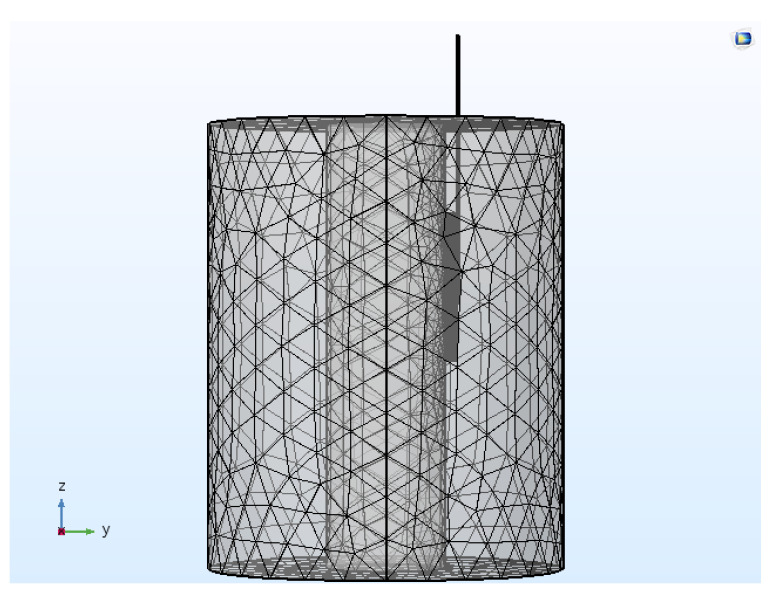
Meshed computational domain of the continuous RF procedure comprising of 174,486 tetrahedral elements.

The discretization of the computational domain was done by utilizing the heterogeneous tetrahedral mesh elements constructed with COMSOL’s built-in mesh generator. An extra refinement close to the active tip of the electrode was applied, where the highest electrical and thermal gradients are expected. Further, a mesh convergence analysis was conducted to determine the optimal number of mesh elements that would result in a mesh-independent solution. Figure 2 presents the meshed computational domain of the heterogeneous model comprising of 174,486 elements and 476,384 degrees of freedom. All the numerical simulations were conducted on a Dell T7400 workstation with Quad-core 2.0 GHz Intel^®^ Xeon^®^ processors. 

### 2.4. Model Validation

**Figure 3:**
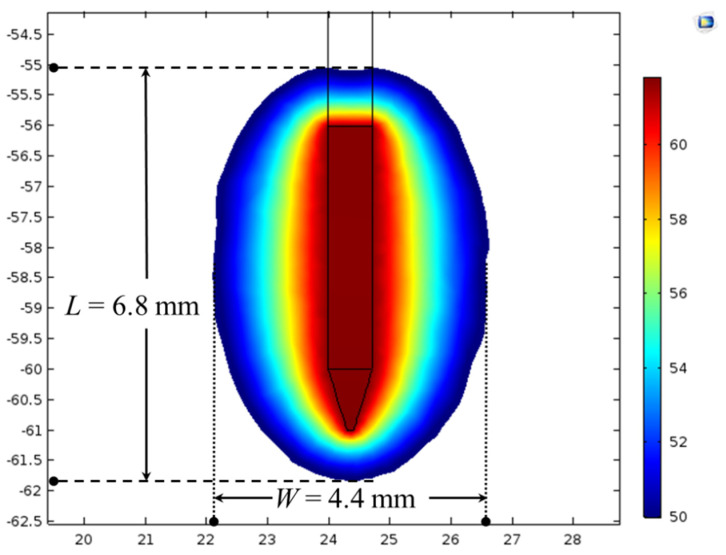
Temperature distribution (in °C) along with lateral (*W*) and longitudinal (*L*) dimensions of the ablation zone obtained after 60 s of continuous RF procedure with the applied voltage of 13 V and an ambient temperature of 37 °C.

The developed computational model’s fidelity and integrity were evaluated by comparing the simulated results of the present model with that of previously reported experimental and numerical findings reported in [45]. All the material properties, dimensions, initial and boundary conditions were considered similar to those of [45]. Table 2 presents the comparative analysis of the rise in temperature (*ΔT*, i.e., the difference between the attained temperature after 120 s of RF procedure and the ambient temperature), that was simulated/computed from the present numerical study to that of the experimental and numerical findings of [45]. As presented in Table 2, the rise in temperature (*ΔT*) predicted by the present numerical study after 120 s of RF procedure for different considered cases was found to be in reasonable agreement with the experimental and numerical findings of [45]. Furthermore, Figure 3 presents the predicted temperature distribution from the current model after 60 s of RF procedure at the applied voltage of 13 V and the ambient temperature of 37 °C and utilizing the same material properties to those of [45]. The outer periphery of the temperature distribution presented in Figure 3 represents the ablation volume quantified using the 50 °C isotherm, and its shape agrees well with the existing knowledge about continuous RF procedure [45]. Importantly, the lateral (*W* = 4.4 mm) and longitudinal (*L* = 6.8 mm) dimensions predicted from the present model were found to be in good agreement with those of [45], which were reported to be around 4 mm and 7 mm, respectively. Additionally, the longitudinal extension of the ablation zone dimensions (corresponding to 50 °C isotherm) in the forward direction from the tip point of electrode and in backward direction from the insulation margin was found to be about 1 mm, analogous to that presented in [45]. 

## 3. Results and Discussion

**Figure 4:**
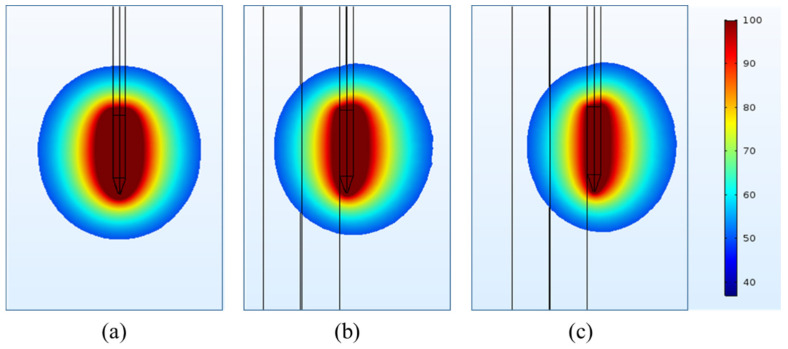
Temperature distribution (in °C) obtained after 60 s of continuous RF procedure in a plane along the electrode axis for: (**a**) homogeneous domain comprising of muscle tissue alone, (**b**) heterogeneous domain comprising of the target nerve embedded in the muscle tissue and (**c**) heterogeneous domain comprising of target bone, nerve and muscle tissues.

**Figure 5:**
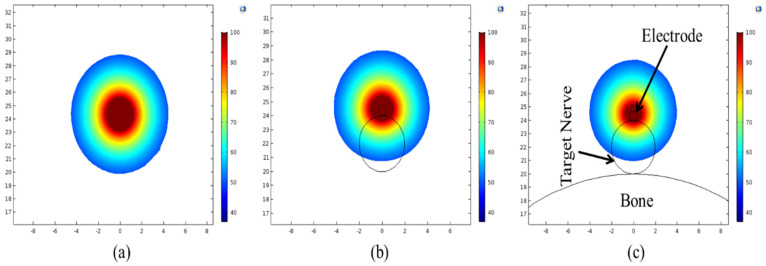
Temperature distribution (in °C) obtained after 60 s of continuous RF procedure in a plane normal to the electrode axis for: (**a**) homogeneous domain comprising of muscle tissue alone, (**b**) heterogeneous domain comprising of the target nerve embedded in the muscle tissue and (**c**) heterogeneous domain comprising of target bone, nerve and muscle tissues.

One of the key objectives of the present numerical study is to quantify the effect of heterogeneous surroundings in the computational domain on the efficacy of continuous RF procedure for pain relief. In pursuing this goal, the variations in temperature distribution and ablation volume were computed for the homogeneous domain (comprising of only muscle tissue) and the heterogeneous domain comprising of: (a) nerve and muscle tissues, and (b) bone, nerve and muscle tissues. Figure 4 and Figure 5 present the variation in the temperature distribution for different cases considered here obtained after 60 s of continuous RF procedure in a plane along the electrode axis (i.e., front view of Figure 1) and in a plane normal to the electrode axis (i.e., top view of Figure 1), respectively. As is evident from Figure 4 and Figure 5, the uniformity of the temperature distribution is ruined for the cases where nerve and bone are modeled within the muscular domain. 

**Figure 6:**
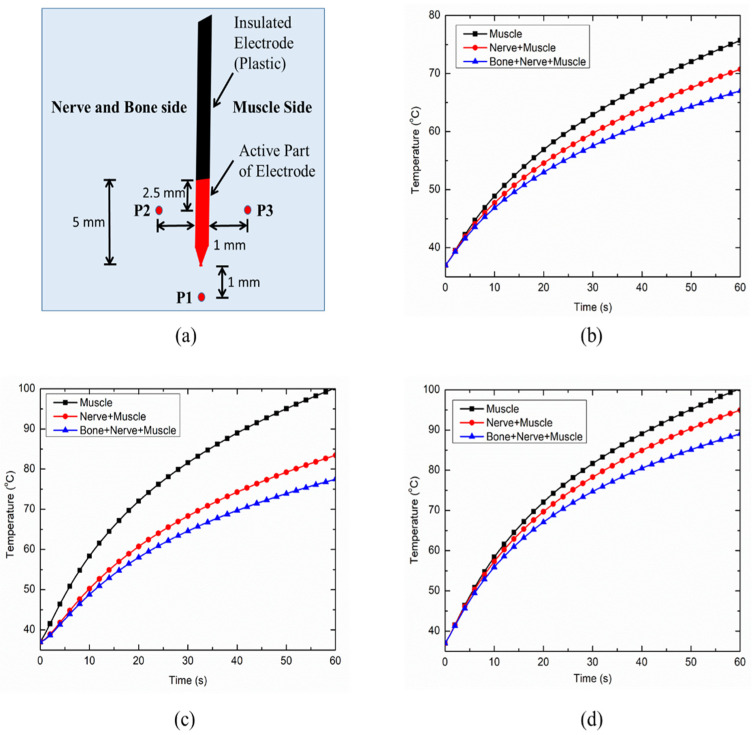
(**a**) Schematic of three points (P1, P2 and P3) chosen for the evaluation of thermal performance during the continuous RF procedure. Variation in temperature distribution as a function of time at: (**b**) P1, (**c**) P2 and (**d**) P3, for different cases considered in the present study.

Three points of interest (as shown in Figure 6a) were defined to analyze the thermal performance of the continuous RF application for treating pain. Importantly, point P1 lies 1 mm away from the tip of the electrode along the electrode axis, while P2 and P3 lie 1 mm away in a transversal plane in the opposite direction from the middle of the active part of the electrode. It is noteworthy that point P2 lies on the nerve and bone side of the computational domain of the heterogeneous model, whereas point P3 lies on the side surrounded by muscles alone. Figure 6b presents the variation in temperature with respect to time at point P1 for the considered cases. As is evident from Figure 6b, the introduction of heterogeneity within the computational domain results in a significant decrease in the predicted temperature at a particular instance. The predicted temperature at point P1 at the end of 60 s of the continuous RF procedure was found to be 75.70 °C, 70.73 °C and 67.03 °C for the homogeneous muscle domain, the heterogeneous muscle and nerve domain, and the heterogeneous muscle, nerve and bone domain, respectively. The variations in the temperature distribution with respect to time at P2 and P3 points are presented in Figure 6c,d, respectively. Again, a significant decrease in the predicted temperature with the introduction of heterogeneity in the computational domain can be clearly seen in these figures. This variation in the predicted temperature during the continuous RF procedure can be attributed to differences in the thermo-electric parameters of the muscle, nerve and bone tissues, as summarized in Table 1. The introduction of the nerve in the homogeneous muscle domain results in the lower electrical conductivity that leads to a distorted electrical field distribution. In turn, this results in lower volumetric heating as compared to the completely homogeneous computational domain comprising of muscles alone. Furthermore, the introduction of bone, which has lower electrical and thermal conductivities as compared to muscle tissue, does not allow efficient heat conduction from the electrode during the continuous RF procedure for treating pain.

**Figure 7:**
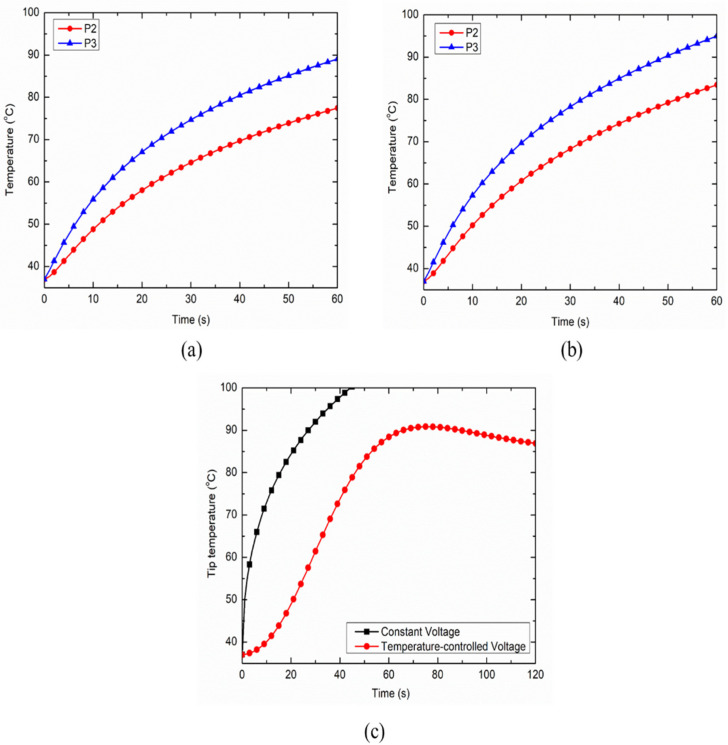
Variation in temperature distribution as a function of time at points P2 and P3 for the heterogeneous computational domain comprising of: (**a**) nerve, bone and muscle tissues, and (**b**) nerve and muscle tissues. (**c**) Variation in the target tip temperature as a function of treatment time for constant voltage and temperature-controlled protocols of power delivery during the continuous RF procedure for pain relief.

The inclusion of nerve and bone within the muscle tissue domain significantly hampers the thermal and electrical performances of the continuous RF procedure for pain relief. Figure 7a,b represent the temperature distribution at points P2 and P3 for the case of heterogeneous computational domains of the muscle, i.e., including both nerve and bone, and including only nerve, respectively. The inclusion of the nerve and/or bone within the homogeneous computational domain of the muscle (for simulating realistic anatomical situations) on one side of the RF electrode restricts the efficient conduction of the heat on that side owing to the lower thermal and electrical conductivities of bone and nerve in comparison to the muscle tissue. These variations result in asymmetric deviation in the temperature distribution on two equally spaced but oppositely placed points P2 and P3 on the transverse axis from the middle of the active part of the electrode, as shown in Figure 7a,b. Note that, for the homogeneous computational domain comprising of the muscle tissue, the temperature profiles at both points P2 and P3 are symmetric and completely coincide with each other. However, the decrements in temperature at points P2 and P3 after 60 s of continuous RF procedure were found to be 12.15% and 13.02% for the heterogeneous computational domain of muscle tissue embedded with only nerve, and embedded with both nerve and bone, respectively. Thus, consideration of the heterogeneous surroundings within the computational model significantly affects the thermal performance of continuous RF procedures for pain relief. 

Further, the comparison between the electrode tip temperature for the constant voltage and temperature-controlled protocols of power delivery during the continuous RF procedure are presented in Figure 7c. It is noteworthy that the temperature sensor for monitoring and maintaining the target tip temperature during the temperature-controlled RF procedure was located at a distance of 1 mm beneath the tip of the electrode, which was inspired by [27]. As evident in Figure 7c, the constant voltage source can result in the attainment of a 100 °C temperature close to the active tip of the electrode, which can then result in charring. This is a highly undesirable phenomenon during RF procedures that leads to an abrupt decline in the electrical and thermal conductivities of the biological tissues, limiting efficient conduction of thermal energy and thereby reducing the ablation size. Thus, the utilization of temperature-controlled power delivery protocol during continuous RF procedures in clinical practices can completely mitigate the chances of charring occurrence, whereby the applied voltage is varied between the electrodes to keep the maximum temperature at the tip of the electrode as 80–90 °C [28].

**Figure 8:**
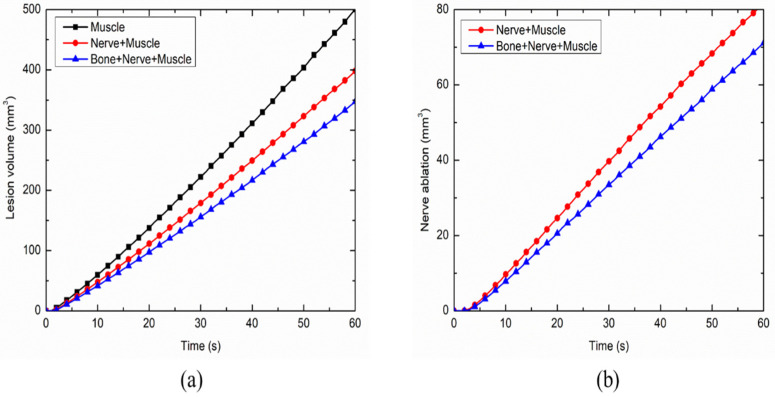
Variations in (**a**) total ablation volume, and (**b**) nerve ablation, with respect to time during the continuous RF procedure for pain relief among different considered cases.

The variation in the total ablation volume obtained for different cases considered in the present study, viz., the homogeneous domain comprising of only muscle tissue and the heterogeneous domain comprising of: (a) nerve and muscle tissues and (b) bone, nerve and muscle tissues, is presented in Figure 8a. As seen from Figure 8a, the attained ablation volume significantly decreases as the heterogeneity in the surroundings is introduced within the computational domain during the continuous RF procedure for pain relief. The propagation of nerve damage with respect to time in the heterogeneous computational domain during the continuous RF procedure is presented in Figure 8b. The introduction of bone within the computational domain results in a 12.75% decrease in the nerve ablation volume after 60 s of continuous RF procedure. Thus, consideration of the heterogeneous surroundings to realistically replicate the anatomy becomes of utmost importance for accurately predicting the treatment outcomes of the continuous RF procedures for pain relief. 

## 4. Clinical Applications, Future Outlook and Model Developments

In this section, we highlight the recent clinical studies related to the application of RF procedures in treating pain. Future research directions for the refinement of the proposed models by utilizing coupled multiscale and multiphysics models are also discussed.

### 4.1. Heterogeneous Surroundings and Clinical Trials

Radiofrequency ablation has been used for pain management since the mid-1970s [46] and has significantly evolved from a therapy that was mainly employed for mitigating neuropathic pain to one of the most promising and frequently applied techniques in clinical practices for alleviating axial spine and musculoskeletal pain [1,16]. Today, the application of RFA in treating pain has steadily expanded and accepted for the treatment of facet joint, sacroiliac joint dysfunction and osteoarthritis [8,9,10,11,12,13,15,16,17,18,19,20]. However, there are significant contradictions and inconsistencies in the reported clinical results on the efficacy of RF procedures. Most of the clinical studies available in the literature are retrospective studies or case studies limited to reporting high-quality randomized controlled trials [47,48,49,50,51,52,53,54,55]. Furthermore, inappropriate selection criteria and treatment parameters could result in poor treatment outcomes, whereas anatomical variations, which are still not well-established, could limit the accurate interpretation of the obtained results. Thus, additional large-scale clinical studies of RF application in pain management are needed with longer follow-up periods to demonstrate its efficacy along with quantification of associated long-term adverse effects. 

There has been continuous development in RF delivery systems and protocols in the quest to increase the ablation volume, which will lead to enhanced efficacy of the RF procedure. Continuous RF is the conventional form of energy delivery, and it causes a decline in the transmission of pain signals by damaging both sensory and motor nerve fibers [1,19]. Three different types of electrodes are typically utilized for delivery of RF energy to the target neural tissue, viz., monopolar, bipolar and cooled RF electrodes (arranged in ascending order of ablation volume generation). Another mechanism of RF energy delivery to the target nerve is the pulsed RF procedure. As mentioned earlier, contrary to the continuous RF that causes tissue destruction by heat, the pulsed RF is virtually painless and does not lead to any neural coagulation and irreversible tissue damage. Instead, it leads to an alteration in the pain signal transduction of the nerve fibers [1,19]. Although the efficacy of pulsed RF has been well-documented, the exact mechanism of action in the mitigation of pain is not fully understood yet [14,18,54,56,57,58,59]. Pulsed RF has shown promise in treating neuropathic pain and certain other clinical conditions where continuous RF is potentially hazardous, such as radicular pain, headaches, chronic shoulder pain, knee and hip pain, axial low back pain, and peripheral nerve pain [18,56,57]. However, further clinical studies are required to quantify the exact mechanism responsible for pain mitigation and broaden the clinical utility of these interventional pain procedures. 

### 4.2. Multiscale Models for Biological Tissues

In order to capture the nuances of the biological cells/tissues exposed to external forces, a multiscale modeling approach provides an efficient and cost-effective alternative for bridging the different scales during the computational analysis [60,61,62,63]. Thus, further refinements in the proposed pain management models can be attained by using multiscale modeling approaches, whereby the damage caused due to RF currents can be quantified at a cellular level. This can be accomplished by coupling predominant phenomena during RF procedures that occur at different scales. Such multiscale models would not only assist in a better understanding of the pre-existing bio-physical behavior during pain management therapy, but also help in predicting the mechanisms that remain elusive to date and in generating new hypotheses for quantifying small-scale effects. Furthermore, while dealing with the small-scale effects of biological cells exposed to RF currents, the development and usage of stochastic models is practically unavoidable [62,64,65]. Moreover, bones in general, and cortical bones in particular, are biological tissues considered as part of our computational domain in the present study. They may exhibit additional effects, based on coupled phenomena, that would be useful to incorporate in further developments of the presented model. Among these, piezoelectricity plays a special role (e.g., [66]). For example, for cortical bones, piezoelectric properties are often responsible for the coupling between macroscopic and micro/nanoscopic scales, which may provide additional insight into certain dysfunctions and diseases [66]. Such properties also provide a foundation for wider usage of these biomaterials in tissue engineering [67]. In describing piezoelectricity, we couple electrical and mechanical fields. The well-posedness of such models of coupled piezoelectricity, along with rigorous energy bounds, were derived by one of us in a series of earlier papers, e.g., [68]. This was done for the first time in a general, dynamic setting through the application of the Faedo–Galerkin procedure and generalized solution technique. Coupled electro-mechanical models have been developed and used in a wide range of applications [69,70,71,72,73,74,75,76,77].

Thermal field treatment requires special attention for problems like those considered in this study. Notably, the thermal effects in this study were quantified considering the classical Fourier’s law of heat conduction with the Pennes bioheat equation as presented in Equation (5), which assumes the infinite speed of heat propagation. However, in biological tissues that have non-homogenous inner structures, accounting for a finite speed of thermal disturbances becomes important, suggesting the existence of non-Fourier conduction with a delay ranging from 10 to 20 s [38,78]. Several studies have been reported providing a way to incorporate such non-Fourier heat transfer behaviors in their computational models, e.g., [23,38,79,80,81,82]. Moreover, the attainment of elevated temperatures during RF procedures can also result in thermo-elastic deformation, including thermal expansion and tissue shrinkage, which is interlinked with many complex small-scale effects, such as protein denaturation, collagen contraction and dehydration. Again, the exact mechanism of such associated effects at an elevated temperature within the biological tissue during thermal therapies are not completely elucidated yet, but significant recent developments have been devoted to this area of research utilizing both experimental and computational studies [23,78]. From a computational perspective, the coupling between thermal and mechanical fields, e.g., for elastic tissues such as muscles, etc., can be done by the development of coupled models of thermoelasticity, as well as efficient numerical methods for their solution, e.g., [83,84,85,86,87,88,89,90,91,92,93,94,95]. Moreover, the development of such models also includes complex nonlinear cases where numerous advances have been made in the improvement of numerical methodologies, e.g., [96,97,98,99]. The coupling of the thermoelasticity and piezoelectric model, as is the case for bone tissues, can be done by the development of piezothermoeleastic models [100,101,102]. This could lead to the development of fully coupled thermo-electro-mechanical models of thermal therapies [23]. Also, the development of such models is relevant to other areas of application, as well as in the development of new methods [103,104]. Furthermore, the proposed model of RF application for mitigating pain presented in this study assumed the quasi-static approximation of Maxwell’s equations (see Equation (1)), whereby the extent of variation of electric and magnetic fields is negligible. Consequently, the electromagnetic field is modeled only by considering the electric field component (neglecting the magnetic field effects) because the wavelength of the electromagnetic field at RF frequency of around 500 kHz is several orders of magnitude larger than the size of the active electrode. However, models exist in the literature that consistently include the magnetic field effects, e.g., [103]. The electrode-tissue and plastic-tissue interfaces (as presented in Figure 1) can be more rigorously modeled by incorporating electrode-tissue contact force [60,105,106,107,108,109], as well as other non-trivial effects, e.g., thermal degradation, spiking, etc., in polymeric materials [110,111,112]. 

Several studies have been reported in the literature for modeling blood perfusion within biological tissues, at both micro- and macro-vascular levels [78,113,114,115,116,117,118,119]. Notably, micro-vascular perfusion refers to the perfusion at a capillary (or small-scale) level while macro-vascular perfusion is associated with the heat-sink effect caused by large blood vessels [120]. The blood flow at a micro-vascular level within the biological tissue is typically modeled by utilizing the porous media theory, whereby the tissue is assumed to be comprised of two phases: the solid phase comprising of cells and the extracellular space, and the fluid phase comprising of capillary size blood vessels [94,95,118,119,121,122,123]. The blood flow within the large blood vessels ( 2 mm in diameter) is modeled by additional coupling of the fluid flow model with the proposed thermo-electric model presented in this study, whereby the geometry of the blood vessel within the computational domain can be incorporated either by including a cylinder or a vascular tree [113,114,115,116,117,124,125]. It is expected that further refinement of the model can be done by deriving the computational domain from actual patient-specific data, which will provide more rigorous analysis and would help medical practitioners to obtain more accurate and precise predictions of the treatment outcomes during the RF application in pain management. Thus, the coupled multiscale framework could assist us in quantifying the unknown biologically-relevant phenomena occurring at cellular and sub-cellular scales and lead to a better understanding of the associated intricacies of RF application in pain management. 

### 4.3. Coupling Frameworks and Pain Relief Models

The application of machine learning has been growing rapidly in the biological, biomedical and behavioral sciences. Importantly, both machine learning and multiscale modeling complement each other in creating more robust predictive models in the current field of research [126,127]. Recently, several studies have been reported in the literature that have explored the application of AI and machine learning algorithms in the field of thermal therapies [40,128,129,130,131,132,133,134,135,136]. The integration of machine learning with the coupled models could play a vital role in decision-making processes and the treatment planning stage of such procedures, e.g., by providing a priori information about electrode placement for enhancing treatment efficacy or by the real-time monitoring of the damage to the target tissue and other critical structures. Furthermore, a general framework of incorporating human factors into mathematical models of complex systems with control has been provided in [137,138]. This can be useful in the context of AI and the machine learning algorithms mentioned earlier in Section 2. Moreover, there has been a surge in the development of neural tissue models for capturing the transduction, transmission, perception and modulation of pain at molecular, cellular and neuron networks levels [78,139,140,141,142,143]. The aforementioned coupled multiscale thermo-electro-mechanical model can be readily integrated with the Hodgkin–Huxley neural model for predicting the treatment outcomes in terms of decline in the actual pain signals that can be coupled with the damage model presented in Equation (7). Such coupling of the neuronal models with the proposed model would assist in our better understanding of the molecular changes affecting the neuronal behavior, such as in quantifying the exact damage to the axons during the application of RF procedures for treating pain. Future studies can be conducted by incorporating the actual physiological neuronal geometries and modeling of biophysical phenomena at sub-cellular scale, viz., accounting for changes in the concentrations of potassium, sodium, calcium and magnesium at the membrane layer [78,144,145,146,147,148]. Such multiscale, multiphysics and fully coupled models will provide a better understanding of the molecular changes affecting the neuronal behavior, along with quantification of the mitigation of actual pain signals during RF procedures.

## 5. Conclusions

This work quantified the effects of heterogeneities, such as nerve and bone tissues, on the efficacy of radiofrequency therapies, exemplifying main results on continuous RF procedures for pain relief. Based on the developed coupled mathematical models and their finite element implementations, a comparative analysis was conducted to evaluate the impact of different heterogeneities in the surrounding computational domain on the temperature distribution and the obtained ablation volume. Among other results, it was found that there was a decrease of 30.64% in the attained ablation volume considering a heterogeneous domain comprising of bone, nerve and muscle tissues, as compared to a homogeneous domain of muscle alone. Further, it was concluded that there is significant variation in the predicted temperature distribution among the different cases considered in the present study. Subsequently, this study emphasizes the importance of considering the effect of heterogeneous surroundings on the predicted treatment outcomes of continuous RF procedures for treating pain. It is expected that the results presented in this study will assist pain management clinicians and researchers to better tackle the issue of the variability in thermo-electric and biophysical properties. This can be achieved by proper treatment planning and consideration of the impact of each parameter on the treatment outcomes. The work also critically analyzed possible directions for further improvement of the developed models. This includes the incorporation of the neural model to replicate pain transmission and mitigation during RF procedures for pain relief. In addition, patient-specific models can be developed and integrated into the clinical workflow to quantify a priori estimates of the treatment outcomes and the risks involved during such minimally invasive treatment procedures.

## Figures and Tables

**Table 1:** Thermo-electric and biophysical properties of different materials considered in this study.

Material (Tissue/Electrode)	Electrical Conductivity *σ* [S/m]	Specific Heat Capacity *c* [J/(kg·K)]	Thermal Conductivity *k* [W/(m·K)]	Density *ρ* [kg/m^3^]	Blood Perfusion *ω_b_* [s^−1^]
Muscle	0.446	3421	0.49	1090	6.35 × 10^−4^
Bone	0.0222	1313	0.32	1908	4.67 × 10^−4^
Nerve	0.111	3613	0.49	1075	3.38 × 10^−3^
Plastic	10^−5^	1045	0.026	70	–
Electrode	7.4 × 10^6^	480	15	8000	–
Blood	–	3617	–	1050	–

**Table 2:** Comparison of the rise in temperature (Δ*T*) reported by Cosman Jr. and Cosman Sr. [45] in terms of both numerical and experimental findings to that computed from the present model after 120 s of the continuous RF procedure.

Applied Voltage(V)	Electrical Conductivity *σ* [S/m]	Ambient Temperature [°C]	Numerically Predicted Δ*T* from the Previous Study [45] [°C]	Experimentally Measured Δ*T* from the Previous Study [45] [°C]	Δ*T* Computed from the Present Study [°C]
7	0.38	26	6.8	7	6.75
13	0.44	26	27.7	26	26.83
16	0.47	26	44.8	41	43.39
16	0.47	34	44.8	48	43.40
